# A Comparative Study of Life History Traits in *C. briggsae* and *C. elegans*

**DOI:** 10.17912/micropub.biology.001299

**Published:** 2024-11-01

**Authors:** Nikita Jhaveri, Harvir Bhullar, Bhagwati Gupta

**Affiliations:** 1 Biology, McMaster University, Hamilton, Ontario, Canada; 2 Biology, Johns Hopkins University, Baltimore, Maryland, United States

## Abstract

The nematodes *C. elegans* and *C. briggsae* are key models for genetic studies. Despite their overall similar morphology, these two species exhibit notable differences. We used the isolates from tropical ( AF16 and QX1410 ) and temperate ( HK104 and VX34 ) regions to characterize the life history traits of *C. briggsae* . Our findings reveal significant variations in body dimensions, movement patterns, utse morphology, and lipid contents across isolates, highlighting species-level distinctions that further establish *C. briggsae* as a valuable comparative model for genetic research.

**Figure 1. Characterization of various traits in C. briggsae and C. elegans wild isolates:**
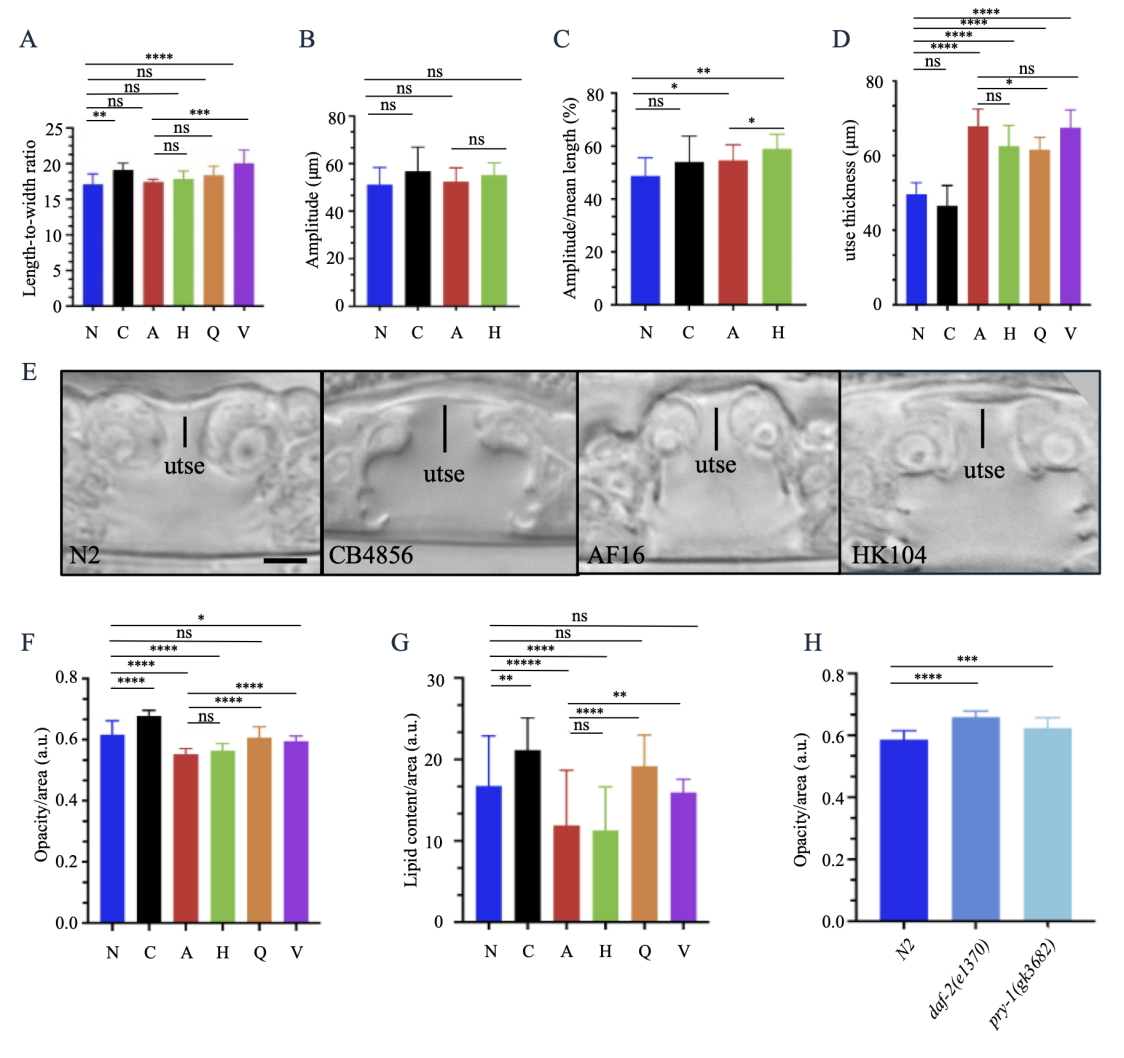
Strains are abbreviated as N (N2), C (CB4856), A (AF16), H (HK104), Q (QX1410), and V (VX34). **A.** Length-to-width ratios of day-1 adult hermaphrodites. Overall, N2 has the smallest ratio and VX34 the largest. Within *C. briggsae* , AF16 has the smallest ratio. The numbers of animals and other details are provided in Table 1. **B, C.** Movement analysis of day-1 adult hermaphrodites. *C. briggsae* strains have amplitudes comparable to *C. elegans* , however the amplitude per unit length shows some differences. n = 8-10 worms for each strain in three or more batches. **D, E.** utse thickness in hermaphrodites at the L4 larval stage. *C. briggsae* isolates have thicker utse than *C. elegans* . n = 8 - 12 worms for each strain, combined from two-three batches. Scale bar 5 mm. **F.** Opacity (measured as pixel brightness) of different isolates, measured in triplicates in day-1 adult hermaphrodites. n = 20 to 30 worms for each strain. **G.** Oil Red O staining of day-1 adult hermaphrodites, done in triplicates with n = 20 to 30 worms for each strain. **H.** Opacity of N2, *daf-2(e1370)* and *pry-1(gk3682)* day-1 adult hermaphrodites. Mutants are darker than N2. n = 17 to 27 animals in a total of 3 batches for each strain. The units are arbitrary (a.u.) in panels F-H. In all graphs, data are shown as mean ± SD. Statistical analyses were performed using one-way ANOVA with Dunnett's multiple comparisons test for interspecies comparisons in panels A-D, F, G. Student's unpaired *t-* test was used for intraspecies comparison in panels A-D, F, G, and also to compare the mutants to N2 in panel H. Statistically significant values are indicated by star (*): * *p* < 0.05; ** *p* < 0.01; *p* < 0.001; **** *p* < 0.0001; ns, not significant.

## Description

To enhance the utility of *C. briggsae* as a genetic model, we characterized its life history traits using two tropical strains ( AF16 and QX1410 ) and two temperate strains ( HK104 and VX34 ). Initial measurements indicated significant variations in body length among *C. briggsae* isolates, with VX34 being the longest and HK104 the shortest. Additionally, *C. briggsae* isolates are generally more slender than *C. elegans* N2 , but not CB4856 (Table 1). To further examine differences in the sizes of isolates, we determined length-to-width ratios of hermaphrodites and found that all *C. briggsae* isolates ranged from 17.1 to 19.7 with VX34 showing the highest ratio ( Figure 1A ). Notably, N2 and AF16 exhibited the smallest length-to-width ratios. Additionally, for some of the strains ( N2 , AF16 , and HK104 ), we examined adult males and observed differences in length and width (Table 1). Overall, these data show that *C. briggsae* and *C. elegans* isolates have considerable variation and suggest significant dimensional diversity across isolates and between species. This variability in size aligns with observations in other nematodes, such as *C. inopinata* and various *Rhabditida* species (Flemming et al., 2000; Hammerschmith et al., 2022) . While the basis for size variations in *C. briggsae* isolates remains to be investigated, studies in other nematodes, including *C. elegans* , have reported the involvement of genetic and environmental factors (Gumienny and Savage-Dunn, 2013; Kammenga et al., 2007; Maulana et al., 2022; Nyaanga and Andersen, 2022; Van Voorhies, 1996) , with specific genes such as the *sma* ( *small* ) class and *tra-3 /Calpain 5* playing crucial roles (Gumienny and Savage-Dunn, 2013; Kammenga et al., 2007) .

The next phenotype that we assessed was the sinusoidal movement patterns. Our preliminary observations suggested differences between N2 and AF16 , so we quantified movement tracks on bacterial lawns in two isolates of each species. The results revealed that while the amplitudes of different isolates are comparable ( Figure 1B ), the amplitude per unit length showed some differences with N2 having the lowest value ( Figure 1C ).

Previous studies noted that the vulva-uterine connection ( ut erine- se am cell, utse) in AF16 was thicker than in N2 (Gupta and Sternberg, 2003) . We found that other *C. briggsae* isolates exhibit a similar phenotype, with the utse being approximately 50% thicker than both N2 and CB4856 animals ( Figure 1D, E ). In spite of this difference, there was no obvious impact on egg-laying frequency and brood size. Further experiments are needed to determine whether this trait affects egg-laying behavior in the two species.

Among other characteristics, it was noted that AF16 adults are lighter in color than N2 . To follow up on this observation, we measured the transparency of adult hermaphrodites and found that *C. briggsae* strains are generally more transparent than *C. elegans* , with variability across isolates ( Figure 1F ). To determine if fat levels were affecting the body color, we carried out Oil Red O staining, which has shown to be a true representation of stored fat content, and positively correlates with the levels of triglycerides (Yen et al., 2010) . The results revealed comparatively lower lipid levels in some *C. briggsae* isolates but the pattern was inconsistent ( Figure 1G ). Interestingly, mutations known to affect lipid content in *C. elegans* (high in *daf-2* mutants and low in *pry-1* mutants) (O'Rourke et al., 2009; Ranawade et al., 2018) also resulted in increased opacity ( Figure 1H ). The results lead us to conclude that while lipids may affect opacity, other factors also contribute to differences in body color.

The above results broaden our understanding of *C. briggsae* as a genetic model and its distinguishing features from *C. elegans* . The results add to the existing body of work documenting differences between the two species that include excretory duct placement (Wang and Chamberlin, 2002) , arrangements of bursal rays in the male tail (Fitch, 1997; Fitch and Emmons, 1995) , P3.p vulval precursor competence (Delattre and Felix, 2001) , systemic RNAi (Winston et al., 2007) , resistance to viral infections (Felix et al., 2011; Franz et al., 2012; Frezal et al., 2019), electrotaxis (Rezai et al., 2011) , and dauer formation (Inoue et al., 2007) . Collectively, these findings contribute to ongoing comparative studies and underscore the importance of species-specific traits in genetic and developmental research.

**Table 1.** Measurements of one-day-old adult animals. Values are shown as mean +/- SD (Standard deviation). N, Number of animals examined. Statistical analysis was carried out using one-way ANOVA using Dunnett's multiple comparisons test. In the case of AF16 and HK104 males, length and width were analyzed using Student's *t* -test. The *p* value columns show statistical comparisons. Isolates used for pair-wise comparison are in brackets where ‘h' denotes hermaphrodites and ‘m' denotes males. Significant values are indicated by stars: * *p* < 0.05; ** *p* < 0.01; *p* < 0.001; **** *p* < 0.0001. ns, not significant.

**Table:** 

**Strain**	**Length (μm)**		**Width (μm)**		**Length-to-width ratio**		N
	Mean +/- SD	*p* value	Mean +/- SD	*p* value	Mean +/- SD	*p* value	
N2 hermaphrodites	1051.3 +/- 81.4	-	61.5 +/- 2.6	-	17.1+/-1.5	-	18
CB4856 hermaphrodites	1002.6 +/- 31.4	ns ( N2 h)	52.7 +/- 3.8	**** ( N2 h)	19.1+/- 1.0	** ( N2 h)	15
AF16 hermaphrodites	961.4 +/- 21.4	** ( N2 h)	55.2 +/- 1.4	* ( N2 h)	17.4+/-0.4	ns ( N2 h)	10
HK104 hermaphrodites	934.6 +/- 47.6	*** ( N2 h), ns ( AF16 h)	52.7 +/- 3.6	**** ( N2 h), ns ( AF16 h)	17.8+/-1.2	ns ( N2 h) ns ( AF16 h)	10
VX34 hermaphrodites	1076.0 +/- 93.3	ns ( N2 h), ** ( AF16 h)	54.6 +/- 2.2	** ( N2 h), ns ( AF16 h)	20.0+/- 1.9	**** ( N2 h), *** ( AF16 h)	13
QX1410 hermaphrodites	1023.0 +/- 94.2	ns ( N2 h), ns ( AF16 h)	55.7 +/- 3.6	* ( N2 h), ns ( AF16 h)	18.4 +/- 1.3	ns ( N2 h), ns ( AF16 h)	16
N2 males	968.2 +/- 20.3	-	49.9 +/- 3.0	-	-	-	10
AF16 males	795.7 +/- 40.6	**** ( N2 m)	38.5 +/- 2.6	**** ( N2 m)	-	-	10
HK104 males	842.2 +/- 43.9	**** ( N2 m), * ( AF16 m)	41.2 +/- 1.9	**** ( N2 m), * ( AF16 m)	-	-	10

## Methods

Worms were cultured on NG-Agar plates using standard methods (Brenner, 1974) . Cultures were maintained at 20°C, which is an optimum temperature for growth, fecundity, and other characteristics of *C. elegans* and *C. briggsae* . Plates were seeded with *E. coli* OP50 as the bacterial food source (Stiernagle, 2006) . For Nomarski differential interference contrast (DIC) imaging, live animals were anesthetized with 1 mM sodium azide and mounted on 5% agar pads on glass slides. The slides were examined using Nikon Eclipse 80i and Zeiss Apotome microscopes. Images were captured using Nikon and Zeiss Zen 3.0 software. For each assay, multiple biological replicates of isolates were processed on different days.

Day-1 adult hermaphrodites were measured using Zeiss Zen 3.0 software attached to a Zeiss Nomarski microscope. L4-staged worms were picked 24 hours prior to analysis and incubated overnight at 20°C on OP50-seeded plates. Measurements of body length and width were performed on young adult hermaphrodites the following morning.

To quantify the amplitude of sinusoidal movement, individual worms were allowed to move freely on NG-Agar plates seeded with an overnight-grown OP50 bacterial lawn. The distance between the peak and trough of the sine wave produced by the worm's movement was measured. The amplitude was calculated as half of this distance. At least one sine wave per worm was analyzed. Additional details on sample sizes are provided in the figure legend.

utse thickness was measured in L4 larvae of hermaphrodites, with the width determined at the center of the hymen region. Opacity (optical density) was measured in day-1 adult hermaphrodites using Nomarski microscopy on anesthetized animals. Lipid content was quantified following fixation and Oil Red O staining of day-1 adults, according to a protocol published earlier (Ranawade et al., 2018) . ImageJ ( https://imagej.net/ ) software was used for image analysis. Worm outlines were traced, and pixel intensities and areas were measured to assess opacity and lipid levels.

## Reagents

**Table:** 

**Strain**	**Genotype**	**Source**
N2	Wild-type *C. elegans*	*Caenorhabditis* Genetics Center
CB4856	Wild-type *C. elegans*	Sternberg lab
CB1370	*daf-2 ( e1370 ) *	*Caenorhabditis* Genetics Center
VC3710	*pry-1 ( gk3682 ) *	Gupta lab
AF16	Wild-type *C. briggsae*	*Caenorhabditis* Genetics Center
HK104	Wild-type *C. briggsae*	*Caenorhabditis* Genetics Center
VX34	Wild-type *C. briggsae*	Andersen lab
QX1410	Wild-type *C. briggsae*	Andersen lab
